# An Overview on the Anticancer Activity of *Azadirachta indica* (Neem) in Gynecological Cancers

**DOI:** 10.3390/ijms19123898

**Published:** 2018-12-05

**Authors:** Marius Alexandru Moga, Andreea Bălan, Costin Vlad Anastasiu, Oana Gabriela Dimienescu, Carmen Daniela Neculoiu, Claudia Gavriș

**Affiliations:** 1 Department of Medical and Surgical Specialties, Faculty of Medicine, Transilvania University of Brasov, 500019 Brasov, Romania; moga.og@gmail.com (M.A.M.); dr.andreeabalan@gmail.com (A.B.); claudiagavris@yahoo.com (C.G.); 2 Department of Fundamental, Prophylactic and Clinical Sciences, Faculty of Medicine, University Transilvania Braşov, 500019 Brasov, Romania; dana_nec@yahoo.com

**Keywords:** *Azadirachta indica*, carcinogenesis, cervical cancer, ovarian cancer, breast cancer

## Abstract

In recent years, a wide range of studies have pointed out the importance of nutraceuticals as reservoirs of therapeutic compounds for several diseases, including cancer. This study is centered on the role of some nutraceuticals as anticancer agents and on their efficiency in the oncological gynecological field. Gynecological cancers include cervical, ovarian, and breast neoplasia and these are the major causes of morbidity and mortality in the female population. Cervical neoplasia affects sexually active women aged between 30 and 40 years and is considered the second leading cause of death for women worldwide. Epidemiological studies have shown a strong association of this cancer with human papilloma virus (HPV) infection, independent of any others risk factors. Ovarian cancer represents about 4% of all women’s cancers and breast neoplasia registers 52.8 new cases per 100,000 women annually. Since ancient times, herbal therapies have shown a wide range of beneficial effects and a high potential for safeguarding human health. *Azadirachta indica* (Neem) is a medicinal plant of Indian origin, a tree with more of 140 isolated compounds and at least 35 biologically active principles that have shown an important influence as tumor suppressors by interfering with the carcinogenesis process. Used for centuries in Asia as a natural remedy for cancer, neem compounds present in bark, leaves, flowers, and seed oil have been shown to possess properties such as chemopreventive capacity, apoptotic activities, immunomodulatory effects, and induction of p53-independent apoptosis. The current study is a systematic literature review based on the anticarcinogenic potential of neem compounds in gynecological cancers.

## 1. Introduction

Cervical neoplasia is considered the second leading cause of death in women, with approximately 24,400 deaths every year in Europe [1]. Romania was recognized by Globocan 2012 as being one of the top 20 countries in Europe in the ranking of mortality from cervical cancer, with 10.8 deaths per 100,000 every year [2]. According to the World Health Organization (WHO), Romania is also in first place in Europe with 2–2.7-fold higher mortality rates by cervical cancer than other European countries [3], and it is expected that those values are going to increase by 2020, rising to 15 million deaths [2,4]. Several epidemiological studies have illustrated the strong association between the development of cervical neoplasia and human papilloma virus (HPV) infection. Several HPV cancer-related types have been discovered and HPV DNA have been detected in 93% of the tumors [4,5]. The role of HPV infection in the carcinogenesis process is considered to be independent of any other risk factors.

Regarding ovarian cancer, the incidence and mortality rate is also essential to establish. Ovarian cancer is in eighth place among the most common cancers in women, including 4% of all cancers [6]. Each year, approximately 225,000 women are diagnosed with ovarian cancer, and 140,000 women die annually from it. The risk of being diagnosed with this type of neoplasia is 1:71, while the probability of dying from this cause is 1:95 [6,7]. Even the incidence and mortality rates of ovarian cancer are high, and the pathophysiology and etiology of this disease is still not fully known and understood [8,9]. There are three categories of factors involved in the development of ovarian cancer: protective factors (e.g., oral contraceptive consumption), risk factors (e.g., family history of cancer, age), and factors such as age at menarche and at menopause, which are not proven implications in the causality of ovarian cancer [8,10,11,12,13].

According to the 2003 National Cancer Registry Report, malign pathology of breast is one the most common cancer types affecting women of reproductive age, with an incidence rate of 52.8/100,000 [14]. Similar to ovarian cancer, the trigger factors for breast cancer are not fully known, but there are some risk factors mentioned in the causality of the carcinogenesis process, such as early menarche or late menopause and family history. However, up to now, no realistic option for primary prevention of breast cancer could be performed [8]. Regarding to the conversion of normal cells to cancer cells, oncogenes seems to have an important role. Oncogenes are the genes that encode proteins involved in cell cycle regulation and are capable of producing proteins involved in cellular transformation. Abnormally expressed or overexpressed oncogenes can convert normal cells to cancerous cells [15].

In the literature, studies have been conducted in order to highlight the importance of phytochemicals, which are bioactive compounds that can interfere with the carcinogenesis process, with a high potential in cancer prevention and treatment [16]. Phytochemicals are plant bioactive compounds that have been increasingly studied recently thanks to their therapeutic potential. The classification of phytochemicals includes phenolics, terpenoids, fiber, and alkaloids. These phytochemicals can improve health by acting as scavengers, substrates for various biochemical reactions, ligands that antagonize the cell surface or intracellular receptors, cofactors of enzymatic reactions, etc. They have multiple beneficial roles, including prevention of cancer, coronary heart disease, diabetes, antimicrobial, antiviral, antiparasitic, etc. [17].

Regarding the carcinogenesis process, it had been proven that phytochemicals can modify normal cell proliferation and regulation, being involved in a wide range of signaling pathways modified during carcinogenesis [18,19]. Waladkhani [20] reported in a study that the mechanism of action of phytochemicals in neoplasia prevention is varied and is represented by the inhibition or induction of different enzymes: phase I and phase II enzymes, respectively, scavenging DNA reactive agents, preneoplastic lesions suppression, and inhibition of certain properties of cancer cells [21].

The therapeutic actions of plant products or natural products play an important role in disease management and prevention. The efficiency of medicinal plants and natural products is nowadays enthusiastically researched, due to their great properties and less-adverse effects. Also, numerous pharmacologically active drugs are derived from plants [22,23,24]. The important role of herbs and medicinal plants in the management of a large number of diseases is also supported in some religious documents, such as the Bible and Queran, sustaining the use of herbs/fruits in the cure of various diseases [25].

*Azadirachta indica* (Neem) is an evergreen tree that belongs to the Meliaceae family and is found in tropical and subtropical areas, including India, Burma [26], Pakistan, Bangladesh, and Nepal [24]. Active compounds from neem have been applied in Ayurveda, Unani, and homeopathy, and nowadays, modern medicine is still using this “divine tree” [26] for treatment of many diseases, including infections, metabolic diseases, and cancer [27].

*A. indica* contains a wide range of biological active compounds, including nimbin, nimbidin, nimbolide, and limonoids. The first polyphenolic flavonoids from fresh neem leaves were quercetin and β-sitosterol [24]. All of those compounds occupy essential places in cancer development and management, through antitumor activity [28,29], antioxidant activity, and the inhibitory effect exerted on the development of malignant cells by modulation of cellular proliferation, tumor suppressor genes, and apoptosis [30].

This paper is a review of the studies regarding the possible correlation between a decreased risk of gynecological cancers (cervical, ovarian, and breast cancers) and the flavonoid from *A. indica* intake.

## 2. Carcinogenesis and Chemopreventive Activity of Phytochemicals

The term “chemoprevention” is used to express cancer prevention and control by using natural products, herbs, or plants called phytochemicals. The assumption that cancer occurrence may be completely prevented, blocked, or reversed is becoming increasingly widespread [31]. In 1948, Berenblum and Schubik proposed the concept of multistage carcinogenesis, which was supported later in various studies [32].

The changes produced in a normal cell are caused by multiple carcinogenic environmental factors, such as biological, chemical, or physical agents that produce molecular changes, resulting in malignant tumor development [33]. To reach malignant transformation, a normal cell requires a balance between proto-oncogene aberrant activation and tumor suppression gene inactivation [32]. For the conversion of proto-oncogenes to active oncogenes, at least five mechanisms have been described: overexpression of proto-oncogenes following acquisition of a novel promoter; the amplification of gene copies that cause an increasing transcript and gene product; influences on the transcription that causes the amount of gene product; the deregulation of the gene caused by chromosomal translocation that produces the juxtaposition of the oncogene and immunoglobulin domain; and alterations in the oncogene structure [34].

**Figure 1:**
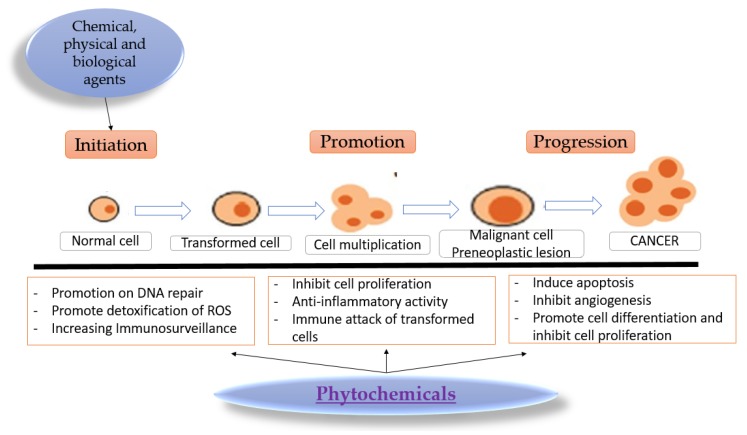
Carcinogenesis mechanism—a multistep process induced by a wide range of chemical, physical, and biological agents and that is interfered with by the protective and anticancer activity of phytochemicals (adapted after Kotecha [36] and Harris [46]).

The main phases of carcinogenesis consist of initiation, promotion, and progression [32]. Figure 1 is a schematic representation of this process, which can convert a normal cell into a premalignant lesion and, finally, into cancer and invasion metastasis. Also, Figure 1 shows the beneficial effects of phytochemicals on chemoprevention, and how the bioactive compounds of phytochemicals are able to block the events of tumor initiation, promotion, and progression.

The first step in neoplasia is initiation, which involves several cellular changes (spontaneously or induced) through a wide range of carcinogen factors. In the first step, the cellular genome undergoes mutations and the potential for neoplasia arises. The second step is represented by promotion, characterized by the stimulation of an initiated cell to undergo further proliferation. The neoplastic conversion of an initiated cell involves multiple steps and requires prolonged exposure to promoting stimuli [35]. This is a reversible process that has, as a result, a range of mutations that lead to a premalignant lesion. The division and propagation of transformed cells begins after promotion [36,37].

This progression is characterized by successive changes arising in a malignant lesion. The molecular mechanism of tumor progression is not fully understood. The implication of mutations and chromosomal aberrations is considered an important factor for tumoral progression. A study by Lala et al. [38] found that the progression of a large number of human tumors could be stimulated by a short-lived molecule called nitric oxide, which is produced by nitric oxide synthetases (NOS). Nitric oxide can stimulate tumor growth and the metastasis process by promoting various specific abilities of tumoral cells, such as migratory, invasive, and angiogenic potentials. So, the selective inhibitors of NOS such as phytochemicals, through their antioxidant activity, can be essential in cancer therapy.

In cervical neoplasia, during the carcinogenesis process, the transforming growth factor β1 (TGF-β1) plays a key role in the development of malignant tumors, the invasion of transformed cells, and the metastasis process. TGF-β1 factor is considered a tumor inhibitor in precancerous lesions, but different mutations or loss of expression of TGF-β1 receptors and SMAD proteins can switch it, and TGF-β1 might be converted into a tumor promoter in later stages of cervical neoplasia. The oncoproteins of human papillomaviruses, which is a promoter of cervical cancer, have been demonstrated to stimulate the expression of TGF-β1, arising in the suppression of the host immune defense [39].

The nuclear factor kB (NF-κB) is an important transcription factor that belongs to a large family of transcription factors and also plays a role in the development and progression of cancer through aberrant activation. NF-κB is essential in modulation of inflammation and immune response, but this function may be altered by abnormal activation. Furthermore, through this mechanism, NF-κB becomes key for proliferation, migration, and apoptosis [40]. Using the immunomodulatory and anti-inflammatory activity of phytochemicals, this step of carcinogenesis could be reversed.

Invasion is the final step of carcinogenesis. As tumor progression advances, malignant cells lose their adherence properties and are transported from the primary organ to other sites, resulting in distant metastasis. After analyzing the epigenetic modifications of tumor suppressor genes in paraneoplastic tissues, carcinogenesis and metastasis processes have been reported to be accelerated by these modifications [41].

Despite advances in medicine, cancer is still a worldwide health problem, and since ancient times, a wide range of plant extracts have been used for treating and preventing cancer. Pure extracted phytomolecules, or nutraceuticals, are functional foods [42] and may help the tumorigenic action of carcinogens by suppressing cell proliferation and mutagenic activity [43]. Nutraceuticals also can raise natural killer cells (NK) function and tumor necrosis factor (TNF α) in patients with late-stage cancer [44]. Other cellular effects of phytochemicals are: the removal of tumoral transformed cells, the increase of immune surveillance, and the prevention of carcinogens to reach targeted sites [45].

## 3. Neem plant (*A. indica*)—Botanical Characteristics and Beneficial Role in Health and Disease Prevention

Neem is a native plant of southeastern Asia, also known as sacred gift of nature, distributed and used for its therapeutic and ethnomedicinal values since ancient times [47,48]. Neem is an evergreen tree that belongs to Meliaceae family, genus *Azzadirachta*. Is also named “Nimba”, “holy tree”, “Vembu”, “Arishtha”, “Indian neem tree”, “Indian lilac”, “wonder tree”, “village dispensary”, “divine tree”, or “panacea of all diseases”. It has a good growth rate and may reach approximately 15–20 m in height, sometimes up to 35–40 m [26].

Neem is highly exploited as a medicinal plant and has been used in different systems, such as Ayurveda, Unani, and homeopathic medicine, and nowadays, it is used by modern medicine [47]. In traditional medicine, different parts of the plant or different extracts have been used, but neem seeds seem to be the most important part of the plant due to their oil content and various bioactive compounds. Also, the seeds have an increased content of azadirachtin, a natural insecticide [49,50]. The lipid content of neem seeds varies from 20% to 32% [51,52], and the content of sterols, fatty acids, and proteins has also been reported. Neem seed oil contains more than 100 active compounds, called triperpenoids and linonoids: saladucin, valassin, meliacin, Nimbin, Nimbicin, geducin, and azadirachtin [53,54], one of the most important biopesticides [55,56].

In their study, Ara et al. [57] showed new tri-(4-8) terpenoidal and di-(9) terpenoidal constituents in neem, and a new tetranortriterpene named nimbilin was discovered. Nimonilin, a tricyclic diterpene, is also a constituent of neem. The seeds seem to be the most important part of neem, but the leaves, fruits, kernel bark, flowers, roots, twigs, and wood may also be used for their beneficial activities.

Biswas have been classified as compounds isolated from neem in two different categories: isoprenoids and nonisoprenoids. Diterpenoids, triterpenoids, and steroids, including azadirone, protomeliacins, limonoids, gedunin, vilasinin, and C-secomeliacins (azadirachtin, nimbin, and salanin), are included in the isoprenoid group. Meanwhile, the nonisoprenoids contain proteins, amino acids, polysaccharides, and polyphenolics (flavonoids, coumarin, tannins, and aliphatic compounds) [47]. The stem bark of neem contains bioactive compounds as tannins: tricyclic diterpenoids, NB-II peptidoglycan, gallic acid, epigallocatehin, gallocatehin, catechin, epicatechin, margolone, etc. [26].

**Figure 2:**
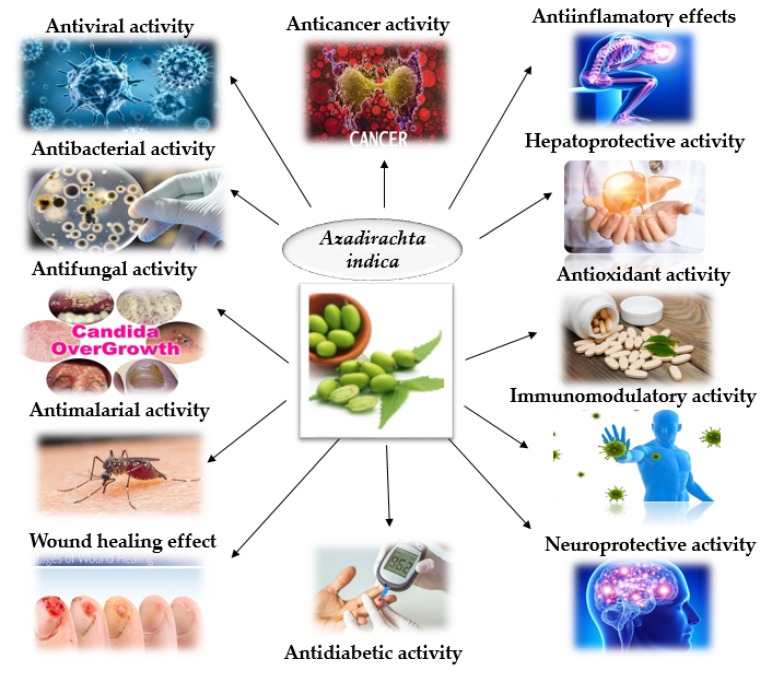
The beneficial activity of neem in general health, disease prevention, and treatment (adapted from Tiwari [26]).

The active principles from bark, leaves, seeds, and other parts of neem are exploited for their proven immunomodulatory, antiseptic, diuretic, antipyretic, antiparasitic, antimicrobial, analgesic, antifeedant, contraceptive, pediculicide, antiulcer, antimutagenic, and anticancer effects [58,59,60]. Figure 2 represents the synthesis of all the beneficial effects of neem extracts.

Regarding the effects on tumorigenesis, the antitumorigenic properties, including immunomodulatory and apoptotic activities, have been exploited since ancient times in prevention and suppression of various types of cancer. Nowadays, there are several laboratory research findings that sustain the potent anticancer effect possessed by neem’s components [61]. All the studies included in our review include different parts of the neem plant, such as seeds, flowers, leaves, and fruits, in order to understand the underlying mechanisms of their anticancer effect [61].

Different extracts of neem have been demonstrated to have protective effects against squamous cell carcinoma. In a study by Sarkar et al. on mice, an aqueous preparation from neem leaves generated an immune response that reacted with carcinoembryonic antigen (CEA), and they concluded that it can be used in the immunotherapy of CEA-positive tumors [62].

## 4. Neem Components and Their Effects on Cancer

Carcinogenesis represents a complex multifactorial process, characterized by multiple steps from precancerous lesions to neoplasia, which modifies a normal cell into a cancer cell with increased invasive and metastatic potential. Cancer cells are characterized by proliferation that cannot be controlled, angiogenesis, resistance to apoptosis, and a marked suppression of the immune reaction against tumor cells [63,64,65]. Tumor cells have a special ability to modulate their environment, which facilitates cell invasion, increases inflammation, and induces angiogenesis [66].

In order to rationalize the complexity of cancer, Hanahan et al. [63] described the biological capabilities, or hallmarks, of a neoplasia. These hallmarks of cancer include resisting apoptosis, sustaining proliferative signaling, avoiding growth suppressor molecules, allowing uncontrolled replicative immortality, inducing vascular formation, activating invasion, and the metastasis process. All these biological capabilities of neoplasms are sustained by instable genomes. Tumors are insular masses constituted by apparently normal cells that possess the capacity of creating a tumor microenvironment, characterized by all these hallmarks.

The important role of neem components in cancer prevention consists of the capacity to modulate the tumor environment through several actions, including decreasing angiogenesis and increasing the toxicity of the cell.

A study by Mahapatra et al. [67] assessed the antiangiogenic actions of the ethanol extract of neem leaves in endothelial cells originating from the human umbilical vein. Their results suggest that the neem leaf extract is capable of regulating the genes involved in cellular development and functions and decreasing the stimulatory effect of vascular endothelial growth factor (VEGF), exerting strong antiangiogenic effects [68].

Neem extracts can be prepared in several ways, with a series of various solvents consisting in diluted alcohol, ether, petrol ether, and ethyl acetate. The majority of the studies based on the efficiency of neem extracts on cancer used different mixtures of neem compounds, and for this reason, the efficiency of individual components is still insufficiently known [69].

Azadirachtin is a major component of neem found especially in neem seeds. It is a secondary metabolite of neem with a complex structure, and because of its complexity, the first synthesis of this metabolite was not accomplished until 22 years after it had been discovered. Azadirachtin is a feeding deterrent for insects and arthropods whose biosynthesis is still not well known, a valuable pesticide, and is also very useful in preventing cancer progression [70].

Nimbolide is found in many parts of the neem plant and was first described in the leaves and flowers. Nimbolide is a triterpene which is used in preventing and treating many diseases but is also useful for its anticancer activity. Many preclinical studies conducted in order to prove the anticancer activity of nimbolide have shown its tumorigenesis and metastasis inhibitor effect. This component of neem is also nontoxic for normal cells and free of side effects that could cause complications. The anticancer effects of nimbolide are related to the suppression of proinflammatory pathways, to increased apoptosis and growth arrest, and to the inhibition of carcinogenic activation as well as induction of enzymes with antioxidant effects [71]. Muhammad et al. [72] conducted a study in order to prove the cytotoxicity of nimbolide in vitro using different cancer cell lines and normal cells. The cells were seeded with nimbolide in various concentrations for 24 and 48 h. The cytotoxic effect of this neem compound was dose and time dependent and it showed a great effect on cancer cells, superior to the mild effect exerted on normal fibroblasts.

Gedunin is a tetranortriterpenoid isolated from neem seed oil with a D lactone ring. In Indian medicine, the active product gedunin has been administered for infectious diseases such as malaria, but recent studies have shown the potential anticancer efficiency of this product against tumor cells in the ovaries, colon, and prostate through regulation of important signaling pathways [73,74].

Gedunin also demonstrated its anticancer activity as a preventive and therapeutic agent in breast cancer. This bioactive compound exerts its function through inhibition of tumoral cells, modulating several heat shock proteins. [73,74,75,76].

Meliatetraolenone, also known as 24,25,26,27-tetranor-apotirucalla-(apoeupha)-6alpha-*O*-methyl, 7alpha-senecioyl(7-deacetyl)-11alpha,12alpha,21,23-tetrahydroxy-21,23-epoxy-2,14,20(22)-trien-1,16-dione, is a new tetranortriterpenoid compound that has been isolated from the fresh leaves of neem, but its anticancer activity needs further investigation. For the moment, only the insecticidal activity of this neem component has been reported [77].

Presently, 35 limonoids isolated from neem seed extracts have proved their cytotoxic activity. The neem limonoids also include:

- azadiradione type 1–15
- gedunin type 16–20
- azadirachtin type 21–24
- nimbin type 25–33
- degraded limonoids 34, 35.

A study by Kikuchi et al. reported that only seven limonoid compounds have been shown to exert anticarcinogenic activity on human tumoral cell lines. These compounds include 3, 6, 7, 18, 28, and 29 genotypes. [78].

**Figure 3:**
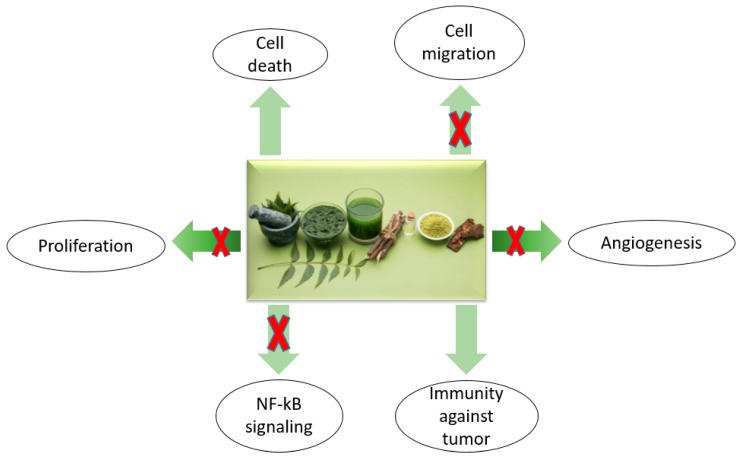
The schematization of neem components as anticancer agents. Their effects include angiogenesis suppression, antiproliferative effects, and suppression of NF-κB. Also, bioactive compounds of neem increase transformed cell death and immunity surveillance (adapted from Hao [69]).

Figure 3 displays the schematized neem compounds that are useful anticancer agents.

Quercetin is a flavonoid from vegetables and fruits that can also be isolated from a 50% ethanolic extract of neem leaves [79]. The structure of quercetin is represented by a basic diphenylpropane C6-C3-C6 skeleton. Even if this flavonoid is consumed daily by people through alimentation, it is now available in dietary supplements known as functional food. After one month of dietary supplementation, the quercetin level in plasma could be increased with 1 g/day and the plasmatic concentration could reach 1.5 µM of quercetin [80].

Quercetin is converted during the intestinal absorption of glucuronide/sulfate conjugates. In circulating blood, quercetin is found only as conjugated metabolites with decreased biological effects, and during inflammation, the enhanced β-glucuronidase activity is able to generate active aglycone from the glucuronide conjugates [81]. After ingestion, quercetin is absorbed by the gut microflora, metabolized in the digestive system, and conjugated to glucuronic acid and methyl/sulfate groups [82]. Regarding the efficiency of this neem compound in cancer prevention, quercetin aglycone seems to alter different signal transduction pathways (Nrf2/keap1 and MEK/ERK) that are linked directly to the processes of carcinogenesis and inflammation. Even if quercetin is a promising anticancer agent, further studies are necessary to elucidate the potency of this neem flavonoid [81].

Epoxyazadiradione (EAD) is an important limonoid found in neem fruits and seeds with cytotoxic activity [83]. Several studies investigated if neem EAD could be used as a cancer prevention agent, and a study by Kikuchi confirmed that it induces early apoptosis in human line 60 cells (observed through flow cytometry). EAD also can induce apoptotic cell death in this type of cell via death-receptor-mediated pathways. Furthermore, this neem compound has high selective cytotoxicity for several leukemia cells [78].

## 5. Studies of Neem as Anticancer Agents in Gynecological Cancers

Neem compounds have shown marked biological abilities and anticancer properties, including: scavenging of free radicals, induction of programmed cellular death, antiangiogenic activity, inhibition of transformed cells proliferation, suppression of the metastasis process and of NF-κB factor, and increase of human immunity surveillance. The suppression of NF-κB is followed by the inhibition of mitogen-activated proteins (MAPKs), protein kinase, and growth-factor-receptor-mediated pathways [84].

Uncontrolled proliferation and lack of transformed cell death are the most important hallmarks in the development of the tumor and metastasis process. So, the inhibition of the continuous proliferation of tumoral cells is the key for many chemopreventive agents.

### 5.1. Cervical Cancer

Cervical cancer is the leading cause of death for women of reproductive age around the world. In order to prove the antiproliferative activity of neem oil obtained from the seeds of *A. indica*, Ricci et al. [68] obtained by methanolic extraction a component of neem oil defined as MEX. The cytotoxic effect of MEX has been estimated in vitro using two cellular lines: a stabilized murine fibroblast line (named 3T6) and a human cervical tumor cell line, HeLA. Their results showed that the tumor cell line (HeLa) had a significantly increased sensitivity compared to 3T6 and that the target of toxicity is the cellular membrane. So, these results imply that MEX could be used as antiproliferative therapy in cervical neoplasia.

Despite the encouraging results of in vitro studies regarding the effects of neem components and extracts as preventive and therapeutic agents of cervical neoplasia, in vivo studies focusing on the beneficial effects of *A. indica* on cancer are still missing. The majority of the investigations made until now used cervical tumor cell lines in order to investigate the mechanisms by which neem extracts can interfere with the induction and progression of cervical cancer.

Another study based on the efficiency of neem and its components on cellular proliferation and on the cell cycle have identified target proteins. Regarding the target proteins in cervical cancer, Priyadarsini et al. [85] designed a study in order to show the molecular and cellular mechanisms of action of azadirachtin and nimbolide as anticarcinogenic agents on the human cervical cancer cell line HeLa. Treatment with nimbolide and azadirachtin decreased the level of cyclin B and cyclin D1 and induced CKI p21 expression, both leading to G0/G1 cell cycle arrest. The suppression of HeLa cell viability functioned in a dose-dependent manner. Also, these two components of neem increased the production of reactive oxygen species by decreasing mitochondrial transmembrane potential and releasing cytochrome c. So, the apoptotic signal is transduced via the mitochondrial pathway.

The release of cytochrome c is regulated by a series of members belonging to Bcl-family, such as Bad and Bcl-xL, respectively, with proapoptotic and antiapoptotic activities [69]. The same study by Priyadarsini et al. mentioned that treatment with nimbolide and azadirachtin induces the modulation of the Bcl-2 family upon exposure to HeLa cells.

Another study made in vitro regarding the antiproliferative activity of ethanolic neem leaves extract on HeLa viability was conducted by Sharma et al. [86]. After treatment with ethanolic neem leaf extract on normal and HeLa cells, cellular growth was differentially suppressed in a dose- and time-dependent manner by apoptosis. The mechanism through which ethanolic neem leaf extract induced cell apoptosis was represented by the modulation of bax, cyclin D1, and cytochrome P450 monooxygenases (CYP 1A1 and CYP 1A2) expression.

In order to demonstrate the potent cytotoxic activity of the aqueous and methanolic extracts of neem, a study performed an immunofluorescence-based apoptosis assay by using a TALI image-based cytometer. The results revealed that 2.68 × 10^6^ out of 2.68 × 10^6^ cells/mL have showed green fluorescence, indicating apoptosis. So, the results indicate that 100% of HeLa cell death was due to apoptosis induced by both aqueous and methanolic extracts of neem [87].

Neem is also mentioned as an efficient immunomodulatory agent in cervical cancer. A study [88] by Roy et al. suggests that neem leaf glycoprotein is a natural immunomodulator that exercises activity on mature dendritic cells, characterized by an increased level of indoleamine 2,3 dioxygenase. In cancer patients, regulatory T cells (Tregs) are amplified and these cells induce overtolerance in dendritic cells and hyperaccumulation of indoleamine 2,3 dioxygenase, which is immunosuppressive. In a culture including both dendritic cells and Tregs isolated from patients with cervical cancer stage IIIB, the neem leaf glycoprotein inhibited the induction of indoleamine 2,3 dioxygenase, limited the expression of cytotoxic T-lymphocyte antigen 4 (CTLA4) on Tregs, and induced normal maturation in dendritic cells. The conclusion of this study was that neem leaf glycoprotein is a promising immunomodulatory agent, but other studies need to be conducted in order to highlight this effect of neem.

Vasenwala et al. [89] used in their study neem fractions to demonstrate how they work on cervical neoplastic tissues removed by biopsy from patients. They also studied the activity of caspase, TNF-α, and IFN-γ levels in monocytes of cervical cancer patients and controls after interaction with neem. The results indicated high enzymatic activity of caspase 3, 8, and 9 in neem-treated monocytes coming from affected patients. TNF-α decreased and IFN-γ increased in the culture supernatant of monocytes, and the cytomorphology of neem-treated cervical cancer cells showed an increased apoptosis level.

The first step in cervical cancer is HPV infection. The role of HPV in cervical cancer, as a risk factor, has been documented and this coexistence is virtually ubiquitous in cervical cancer cases worldwide. There are many studies in the literature which have shown that HPV DNA is detectable in fragments of cervical cancer tissue in almost 90–100% of cases [90]. Neem is also used for its potential anti-HPV activity.

Praneem is a polyherbal alimentary supplement which also contains extracts of neem. An in vivo study conducted by Shukla et al. [91] included 20 women with HPV16 infection who were healthy or ahd low-grade squamous intraepithelial lesions (LSIL). All subjects received topical intravaginal applications of praneem, tablets, or placebo for 30 days, excluding the menstrual period. The results showed a marked elimination of HPV in 60% of cases and also improvement in clinical symptoms and in cytological abnormalities. So, neem is also useful in eliminating the precursor of cervical cancer, HPV infection.

A study by Pryadarsini [92] investigated the mechanism through which quercetin induces HeLa death. Their results showed that quercetin is able to suppress HeLa cell death viability. Quercetin induces apoptosis through a mechanism dependent on p53 and G2/M phase cell cycle arrest in a dose-dependent way. The produced changes included mitochondrial membrane depolarization, upregulation of Bcl-2 proteins with proapoptotic activity, changes in nuclear morphology, and modulation of NF-κB family members and cell cycle regulatory proteins.

Quercetin is also useful as an antimetastatic agent in cervical cancer. Zhang et al. [93] conducted a study in 2009 and their results showed that it can inhibit adhesion, migration, and invasion of HeLa cells and also is able to inhibit breeding of these cells by inducing apoptosis.

Even though the anticarcinogenic effect on cervical cancer of neem’s quercetin is still not completely understood, a study on HeLa cells found that this flavonoid’s antioxidant effect was able to increase AMP-activated protein kinase (AMPK) phosphorylation in order to decrease acetyl-coA carboxylase (ACC) and to suppress these cervical cancer cell lines. Also, it can activate the receptor of epidermal growth factor (EGFR) through the suppression of several phosphatases such as PP2a and SHP-2 and increase the interactions between EGFR and Cbl that are able to induce Cbl tyrosine phosphorylation [94].

### 5.2. Ovarian Cancer

Ovarian cancer is the fifth leading cause of death among women worldwide. The discovery of phytochemicals (neem extracts or neem compounds) as therapeutic agents in ovarian cancer prevention and management is essential because the outcome for patients with this type of cancer needs to be improved.

A very limited number of studies based on the effectiveness of neem plant compounds on the carcinogenesis process in ovarian cancer have been published in the literature. Also, the existing studies have been performed only in vitro on human ovarian tumor cell lines.

The effect of gedunin on the ovarian cell line (SKOV3, OVCAR4, and OVCAR8) proliferation was evaluated by Siddharth et al. [74]. Their results showed a marked cell proliferation decrease after gedunin treatment, up to 80% (*p* < 0.01). They also discovered 52 associated genes, sensitive to gedunin, with major implications for molecular functions associated with the carcinogenesis process, lipid metabolism, and cell cycle control.

The antiproliferative potential of gedunin has also been investigated and evaluated by Tharmarajah et al. [95] on human cells of embryonal carcinoma (NTERA-2, a stem cell model of cancer). The effects exerted by gedunin on the expression of heat shock protein 90 (HSP90), Cdc37 (cochaperone of HSP90), AKT, ErbB2, and HSF1 have been evaluated by polymerase chain reaction. The results indicated that this phytochemical has an encouraging effect on NTERA-2 cells and a reduced influence on primary peripheral blood mononuclear cells (PBMCs). Meanwhile, the expression of HSP90 is inhibited by gedunin, and Bax and p53 are upregulated. The proapoptotic effect of gedunin have been confirmed by the morphological changes associated with apoptosis by DNA fragmentation and the increased enzymatic activity of caspase 3/7.

The inactivation of the Hsp90 cochaperone p23 in vitro by gedunin is another mechanism of this compound. Also, the lethal effect on ovarian cancer cells of the gedunin–p23 complex was investigated by Patwardhan [96], who concluded that this effect is increased by caspase-7-mediated cleavage of the cochaperone, which leads to enhanced apoptotic cellular death.

Nimbolide is found in the flowers and leaves of neem and has a potential cytotoxic effect on human choriocarcinoma. Kumar et al. investigated in vitro this effect of nimbolide using human choriocarcinoma (BeWo) cells. After the cell line treatment with this limonoid, the results showed the inhibition of BeWo cell growth, which was also dose and time dependent. The examination of nuclear morphology also revealed modifications indicating apoptosis. A decreased Bcl-2/Bax ratio associated with increased expression of Apaf-1 and caspase-3 sustain the hypothesis that mitochondrial pathways mediate nimbolide-induced apoptosis [97].

A secondary effect of aqueous neem leaf extract on ovarian tissue is the induction of oocyte apoptosis. Increased granulosa cell apoptosis was proved by Tripathi et al. [98] in a study on rat oocytes. This effect was also accompanied by enhanced expressions of p53 and Bax, decreased expression of Bcl2 protein, increased cytochrome c concentration, and induced DNA fragmentation. The same result was obtained Chaube et al. in their study. They investigated the effect of neem leaf extract on granulosa cells and oocytes in mammals, and based on this study, they proposed that neem leaf extract is able to induce the production of reactive oxygen species and mitochondria-mediated apoptosis, both in granulosa cells and in follicular oocytes [99].

In the literature, there are many studies based on the beneficial effect of quercetin on ovarian neoplasms. Gao et al. [100] investigated this effect in vitro using A2780S ovarian cancer cells. After treatment, quercetin induced apoptosis of A2780S cells associated with increased activity of caspase-3 and caspase-9, downregulation of MCL-1 and Bcl-2, and upregulation of Bax. Also, quercetin decreased phosphorylated p44/42 mitogen-activated protein kinase and phosphorylated Akt. This is the method of contribution to the inhibition of A2780S cell proliferation.

Quercetin is also able to induce growth-inhibitory activity in ovarian cancer cells, OVCA 433 cell line. This activity is possible by modulation of TGF-β1 production [101].

### 5.3. Breast Cancer

Breast cancer is the most common cancer for all ethnic groups among women of reproductive age, with an increased incidence among young women [102]. *A. indica* has been increasingly focused on in recent decades due to its beneficial properties against breast cancer.

Recent reports showed that neem leaf extract is able to induce apoptosis in the MCF-7 breast cancer cellular line [103], and there are many studies which have demonstrated the enhanced effectiveness of ethanolic neem leaf extracts compared to aqueous extracts for cancer treatment [104].

In order to investigate the effect of neem leaf extract on breast cancer, Othman et al. [105] proved that the expression of c-Myc oncogene of BALB/c mice with 4T1 breast cancer cells can possibly be downregulated after ethanolic neem leaf extract administration.

A study by Arumugan et al. [106] was conducted in vivo in order to determine whether the administration of an ethanolic neem leaf extract could inhibit the progression of chemical carcinogen-induced mammary tumorigenesis in rats. After the rats were intraperitoneally injected with *N*-methyl-*N*-nitrosourea and breast tumors appeared, treatment with ethanolic neem leaf extract was administered. Neem extract proved to be highly efficient at reducing mammary tumor volume and at progression suppression. The treatment enhanced genes with proapoptotic effects and also increased Bcl-2-associated death promoter protein (Bad) caspases, p53, phosphatase and tensin homolog gene (PTEN), B cell lymphoma-2 protein (Bcl-2)-associated X protein (Bax), and c-Jun N-terminal kinase (JNK). It also caused downregulation of angiopoietin and vascular endothelial growth factor A (VEGF-A), cell cycle regulatory proteins (cyclin D1, cyclin-dependent kinase 2 (Cdk2), and Cdk4), NFκB, and mitogen-activated protein kinase 1 (MAPK1).

The molecular mechanism of ethanolic neem leaf extract has been also investigated in vitro using two breast cancer cell lines—estrogen-dependent breast cancer cell line (MCF-7) and estrogen-independent breast cancer cell line (MDA-MB-231)—that were exposed to different concentrations of ethanolic neem leaf extract. The results showed that neem leaf increase the level of proapoptotic proteins and decreased the antiapoptotic proteins. Moreover, the bioactive extract induced apoptosis in both cell lines. Another effect of this extract was the decrease of insulin-like growth factor (IGF) signaling molecules IGF-1R, Ras, Raf, p-Erk, p-Akt, and cyclin D1 expression. So, the anticancer activity of ethanolic neem leaf extract is represented by apoptosis induction and its antiproliferative effect, manifested by the inhibition of signaling molecules in MCF-7 and MDA-MB-231 breast cancer cell lines [107].

The antineoplastic activity of neem leaf ethanolic extract has been proven to be more specific at an alkaline pH. Ahmad et al. evaluated the effect of neem leaf extract on human breast cancer cell line MDA-MB-231 at different pH values. They found that a concentration of 1600 μg/mL neem leaf extract at a low pH (6.2) caused the mortality of 95.7% of cells. Meanwhile, at a pH of 7.1, strong cytotoxicity was exerted by a concentration of only 200 μg/mL neem leaf extract. So, although acid pH promotes cancer cell viability, the presence of neem leaf at low pH values potentiates the cytotoxic effect of this phytochemical [108].

Ethanolic neem leaf extracts are not only useful as an anticancer agent. A prospective study by Mandal-Ghosh et al. used an aqueous preparation of neem leaf in order to demonstrate its possible utility in breast cancer immune activation. A breast-tumor-associated antigen (BTAA) with a molecular weight of 85 kDa has recently been identified. The antibodies generated after BTAA and neem leaf aqueous immunization were able to induce cytotoxicity and cytotoxic T-cell response towards BTAA-expressing MCF-7 cells, compared to the antibodies produced by single immunization with BTAA alone, which were able to generate only a slight cytotoxic response. The conclusion of the authors was that the neem leaf extract induces a Th1 response as evidenced by the secretion of IFN-gamma and the greater production of IgG2a antibody in immunized mice [109].

Other fractions of neem used in a rat model were represented by ethyl acetate fraction (EAF) and methanolic fraction (MF). The markers of chemoprevention used by Vinothini et al. in their study on the chemopreventive effectiveness of these neem fractions were represented by estradiol and estrogen receptor status, xenobiotic-metabolizing enzyme activities, redox status, DNA and protein modifications, and the expression of cell proliferation. The administration of a dose of 10 mg/kg MF or EAF was associated with the modulation of hormonal status, modulation of several metabolizing enzymes, enhancement of antioxidants, lipid oxidation, protein modifications, inhibition of oxidative DNA damage, induction of apoptosis, and cellular proliferation. Despite the effectiveness of both fractions, EAF proved to be more effective than MF in the process of modulating different molecular targets [110].

Nimbolide also exerts an anticarcinogenic effect on human breast cancer cells and raises new perspectives for its effectiveness as an anticancer therapeutic agent. The molecular pathway of proapoptotic activity of nimbolide was studied in vitro on MCF-7 and MDA-MB-231 human breast cancer cell lines by Elumalai et al. [111]. The inhibition of MCF-7 and MDA-MB-231 cell growth depended on dose and time of administration, and apoptosis was confirmed by extrinsic and intrinsic apoptotic signaling molecule expression. Also, nimbolide proved to be able to induce the cleavage of pro-caspase-3, pro-caspase-8, and PARP.

Another study that provided evidence that nimbolide has antiproliferative effects on breast neoplastic cells through modulation of the IGF signaling molecules was conducted by Elumalai et al. [112] in 2014. In order to study the interaction between nimbolide and the IGF-1 signaling pathway, the authors used IGF-I and phosphoinositide 3-kinase (PI3K) inhibitor (LY294002) to treat MCF-7 and MDA-MB-231 cells. Their results showed that the expression of IGF signaling molecules was significantly decreased in nimbolide-treated breast cancer cells.

The anti-breast-cancer activity of gedunin has been recently shown to manifest through the inhibition of Hsp90, a protein with a 90-kDa molecular weight. The real molecular mechanism by which gedunin is efficient in breast cancer prevention is still not very well known, but the studies conducted until now in vitro on MCF-7 and SkBr3 breast cancer cells have shown the effectiveness of this natural product [113].

Quercetin is also useful for breast cancer prevention and treatment. A study conducted in order to discover the mechanism of growth inhibition in MCF-7 human breast cancer cells was published by Choi et al. [114]. After 24 h of treatment with quercetin, the authors noticed that the cells specifically accumulated at the G2/M phase of the cell cycle and that cyclin B1 levels and Cdc2 kinase activity increased. After 48 h or longer, Cdk-inhibitor p21CIP1/WAF1 protein level increased and the induction of p21CIP1/WAF1 increased its association with the Cdc2-cyclin B1 complex. So, quercetin is effective in cancer cell growth inhibition through two mechanisms: by apoptosis induction and the inhibition of cell cycle progression and subsequent G2 arrest.

The precise molecular mechanism of quercetin in breast cancer apoptosis is still not completely known. Another study of quercetin on human breast cancer cells concluded that this flavonoid is able to decrease the percentage of viable cells dependent on dose and time of administration, and it also influences the cell cycle arrest and apoptosis process. Reactive oxygen species were not described to be increased, but increased cytosolic Ca2+ levels and reduced mitochondrial membrane potential have been discovered after treatment. The activation of caspase-3, caspase-8, and caspase-9 were promoted by the flavonoid in MDA-MB-231 cells. Quercetin also increased the abundance of Bax protein and decreased the level of antiapoptotic protein Bcl-2 [115].

In order to elucidate the molecular mechanism supporting the anticarcinogenic effect of quercetin in MDA-MB-453 cells, Choi et al. exposed these cells to 100 μM of quercetin for 24 h. After exposure, the authors remarked that quercetin increased the number of sub-G1 phase cells. Qurcetin also increased Bax, cleaved caspase-3, and PARP expression, but reduced Bcl-2 expression was observed [116].

After administering increasing concentrations (12.5, 25, 50, 100, and 200 µM) of quercetin to MCF-7 human breast cancer cells, Duo et al. concluded that at doses of 50–200 µM quercetin are able to inhibit the proliferation of MCF-7 cells. The authors also noticed increased apoptosis after 48 h of exposure (*p* < 0.05). The downregulation of Bcl-2 protein expression and upregulation of Bax expression may underlie the mechanisms behind all the mentioned effects [117].

Table 1 summarizes the studies we found in the literature regarding the antitumoral properties of different compounds and extracts of neem, highlighting the action mechanisms on different types of cells.

Table 2 summarizes in vivo studies that include comparative anticarcinogenic properties of neem fractions and compounds in the oncological field of gynecology.

## 6. Beneficial Combination between *A. indica* Extracts and Classical Anticancer Therapy

Standard therapy in gynecological cancers is usually represented by chemotherapy and radiotherapy, depending on tumoral grading. One of the most serious problems related to these therapies is resistance to chemotherapy and radiotherapy and the secondary effects of chemotherapeutic agents.

Regarding cervical cancer, neither surgical nor radiation treatment strategies have proved to be superior in survival rates. Neoadjuvant chemotherapy has been considered useful in reducing cervical tumor bulk prior to surgery or radiotherapy [118].

For breast cancer cases, neoadjuvant chemotherapy is used in advanced stages of the disease and in inoperable cases to achieve surgical resection and to extend these cases to operable ones in order to facilitate surgery by downstaging [119].

Neem extracts are useful as chemopreventive agents, but their effects combined with standard anticancer therapies are also important to highlight. In the literature, several studies have been conducted in order to prove that neem extracts are able to induce chemosensitization of tumor cells and to reduce the adverse effects and toxicity of these chemical agents.

Kamath et al. [74] conducted an in vitro study in order to prove the efficiency of gedunin on epithelial ovarian cancer, alone and combined with cisplatin. The treatment of SKOV3, OVCAR4, and OVCAR8 ovarian cancer cell lines, realized in vitro by adding gedunin, decreased cell proliferation up to 80%. By combining gedunin and cisplatin and comparing this with cisplatin alone, the authors demonstrated up to a 47% decrease in cell proliferation. So, gedunin is one of the neem compounds which may increase the effect of cisplatin on ovarian tumor cells.

For cervical cancer, it has been proved that ethanolic neem leaf extract is able to enhance the therapeutic power of cisplatin [86]. In vitro treatment of MCF-7, HeLa cells, and normal cells with ethanolic neem leaf extract induced apoptosis on cervical cancer cells, and also, a lower dose combination of ethanolic extract and cisplatin resulted in synergistic growth inhibition compared to the individual drugs. So, we can conclude that neem extract is able to decrease the tumoral transformation of normal cells, alone or combined with chemotherapeutic treatment, while potentiating the efficiency of their efficacy at lower doses.

Regarding breast cancer, Scambia et al. [120] conducted a study in order to prove the additive effect of quercetin on Adriamycin therapy in a multidrug-resistant MCF-7 human breast cancer cell line. The Adriamycin resistance in this cancer cell line is associated with the expression of high levels of P-glycoprotein, and the authors demonstrated that quercetin can reduce the expression of P-glycoprotein in MCF-7 ADR-resistant cells. So, this neem-extracted flavonoid could be a possible anticancer drug either alone or in combination with Adriamycin to potentiate its effect.

Despite the beneficial effect of chemotherapy on cancer, these agents also have adverse effects. Cisplatin is useful as a neoadjuvant agent in cervical cancer, but it can produce nephrotoxicity. Neem is considered to be a promising agent for protection against cisplatin-induced nephrotoxicity. A study investigating the effects of methanolic neem leaf extract on cisplatin induced toxicity of kidneys and increased the level of oxidative stress in rats. After five days of cisplatin injection, the authors observed injuries of the renal tissue (as histopathological damages and increased serum uric acid, urea, and creatinine) and increased levels of nitric oxide. After oral administration of methanolic neem leaf extract for five days, the histological observations evidenced the rescue of the tissue from cisplatin damage and the normalization of nitric oxide products [121].

Another side effect of cisplatin is hepatotoxicity, and neem leaf extract has shown significant protection against this effect when neem supplements are coupled with classical anticancer therapy. Neem’s protective activity was evidenced by the decrease of elevated serum aspartate aminotransferase, alanine aminotransferase, alkaline phosphatase, total bilirubin, urea, uric acid, and creatinine. These findings suggest that neem leaf supplements, administered before, after, or during classical anticancer therapy with cisplatin, is able to prevent hepatic injuries. [122]

Regarding the radiosensitizing effect of neem compounds, this is still not perfectly understood and more studies are needed in order to discover all of the mechanism of radiosensitizing and to overcome the radioresistance of cancer cells. A study by Lin et al. [123] was conducted to analyze the effect of quercetin on different cancer cell lines’ radiosensivity both in vitro and in vivo. Sensitizing enhancement ratios in DLD1, HeLa, and MCF-7 cells were 1.87, 1.65, and 1.74, respectively, and the combination of quercetin and radiotherapy proved to be able to enhance tumor sensitivity to radiotherapy by targeting the ATM-mediated pathway as a response to radiation.

## 7. Toxicity Profile of *A. indica*

Neem has been used by humans for its beneficial effects for centuries and its consumption has been absolutely inoffensive. Neem can be also used in a more concentrated form, as extracts, in order to treat or prevent various diseases, including cancer. The toxicity profile of this phytochemical is outlined according to the solvent used to process the plant in order to obtain concentrated fractions [124].

In animal models (mice), studies have shown an LD_50_ for methanolic leaf extracts of 13 g/kg [125], and for methanolic extracts of *Azadirachta* flowers, the LD_50_ was 12 g/kg [126]. In contrast to the methanolic extract, aqueous neem fractions are considered nontoxic and the LD_50_ is higher than 2.5 g/kg [127].

A study by Tarbousch et al. [128] showed that oral doses of azadirachtin are able to produce adverse effects. Their results indicated that oral administration of this phytochemical did not produce morphological consequences on mouse fetuses. Also, daily administration of 5–50 mg/kg of azadiractin did not produce adverse effects on the reproductive system or on fetus development [129].

In the United States, azadirachtin is considered a nontoxic pesticide. Furthermore, the oil extracted from neem seeds is able to cause death in rats if it is administered intravenously or intraperitoneally and has an LD_50_ of 14 mL/kg [130].

For humans, a daily dose of 15 mg/kg can be toxic, and nonaqueous extracts have demonstrated the presence of allergens in the skin prick test for allergenic activity [124].

## 8. Conclusions

In this paper, we have revealed several neoplasia from the gynecological field that can be prevented and treated using *A. indica* supplementation. This natural agent may not be as effective as conventional chemotherapeutic agents, but its potential in cancer prevention is a fact and has been widely proved by many authors. Also, the use of this phytochemical in cancer prevention is very attractive because neem extracts are widely available, are not expensive, and adverse effects have not been proven.

From the papers that we reviewed in this paper, we concluded that neem tree extracts and compounds have great potential for the prevention of cancer. The molecular mechanism of action involves the modulation of cellular proliferation, differentiation, apoptosis, angiogenesis, and metastasis processes. Up to now, several in vitro studies have been conducted on cancer cell lines, but more in vivo studies are crucial for a better understanding of the beneficial and possibly adverse effects that could be generated by its utilization.

Chemosensitization of tumor cells by using both neem extracts and cisplatin or other chemotherapeutic agents has also gained attention, but the interactions between this plant and classical anticancer therapy should be further analyzed. This combined approach is able to improve the efficiency of standard cancer therapies by allowing for decreased chemotherapy doses, and neem extracts have also proved to be useful in reducing the toxicity of chemotherapy drugs.

In conclusion, this review highlighted the effectiveness of *A. indica* in gynecological cancer prevention and treatment, and we consider that a therapeutic drug from natural sources that is effective in cancer therapy could be a gold mine for both patients and doctors.

## Figures and Tables

**Table 1:** Comparative anticarcinogenic properties of neem fractions and compounds in gynecological cancers based on in vitro studies.

Number	Neem Constituent/Extract	Cell Type	Function	Mechanism of Action	Reference
1	Azadirachtin	HeLa	Cell apoptosis	Enhances CKI p21 expression, decreases cyclin B and cyclin D1 levels, both leading to G0/G1 cell cycle arrest	[85]
			Cell cycle interruption	Induces the modulation of Bcl-2 protein family upon the exposure	
		HeLa	Cell apoptosis and tumor volume reduction	Interacts with cyclin E, causes phosphorylation of the same, prevents the G1/S phase protein expression	[131]
2	Nimbolide	HeLa	Cell apoptosis	Induces the modulation of Bcl-2 protein family	[85]
			Cell cycle interruption	Induces the expression of CKI p21, decreases cyclin B and cyclin D1 level, both leading to G0/G1 cell cycle arrest	
		BeWo	Cells apoptosis and disruption of BeWo cell cycle progression	Decreases Bcl-2/Bax report and increases the expression of Apaf-1 and caspase-3	[97]
		MDA-MB-231, MCF-7	Inhibits cell proliferation (IC_50_ values of 1.97 ± 0.24 and 5.04 ± 0.25 μM), induces autophagy	Reduces Bcl-2, induces Bax and caspases protein expression with modulation of HDAC-2 and H3K27Ac expression. Autophagy signal is induced by increasing Beclin 1, LC3B, and decreasing p62 and mTOR expression	[132]
		MCF-7, MDA-MB-231	Cell apoptosis	Induces the cleavage of pro-caspase-3, pro-caspase-8, and PARP; modulation of the IGF signaling molecules	[111]
3	Gedunin	SKOV3, OVCAR4, and OVCAR8	Inhibits cell proliferation	Up to 80% decrease in cell proliferation	[74]
		NTERA-2	Inhibits cell proliferation	Induces the inhibition of Hsp90, cochaperone Cdc37, and HSP proteins (AKT, ErbB2, and HSF1) and upregulation of Bax and p53	[95]
		SKOV3, OVCAR4, and OVCAR8	Cell apoptosis	Caspase-7-mediated cleavage of the cochaperone p23	[96]
		MCF-7 and SkBr3	Inhibits cell proliferation	Inhibition of Hsp90	[113]
4	Quercetin	HeLa	Cell apoptosis	Induces G2/M phase interruption during the cellular cycle and mitochondrial apoptosis through a mechanism dependent of p53; also induces modulation of NF-κB family members	[92]
		HeLa	Antimetastatic function	Inhibits adhesion, migration, and invasion of tumor cells	[93]
		HeLa	Antioxidant effect	Increases AMPK phosphorylation to downstream acetyl-coA carboxylase. Activates EGFR by suppressing PP2a and SHP-2, and induces the tyrosine phosphorylation of Cbl by increasing the interaction between EGFR and Cbl	[94]
		OVCA 433	Cell-growth-inhibitory activity	Modulation of transforming growth factor β1 (TGF-β1) production	[101]
		MCF-7	Cell-growth-inhibitory activity	Inhibition of cell cycle progression and subsequent G2 arrest	[114]
		MDA-MB-231	Cell apoptosis	Activation of caspase-3, caspase-8, and caspase-9, increasing the abundance of Bax protein and decreasing the level of antiapoptotic protein Bcl-2	[115]
5	Ethanolic/methanolic/aqueous neem leaf extract	HeLa	Cell apoptosis	Modulation of the expression of bax, cyclin D1, and cytochrome P450 monooxygenases (CYP 1A1 and CYP 1A2)	[87]
		HeLa	Cell apoptosis	Intrinsic: cytochrome c, Bcl-2 proteins Extrinsic: death receptors	[86]
		Rats oocytes	Granulosa cell apoptosis	Increases p53, Bax, and p53 expression, decreases Bcl2 expression, increases cytochrome c concentration, and induces DNA fragmentation	[99]
		MCF-7 and MDA-MB-231	Cell apoptosis and cell-growth-inhibitory activity	Decreases the protein expression of insulin-like growth factor (IGF) signaling molecules IGF-1R, Ras, Raf, p-Erk, p-Akt, and cyclin D1.	[107]
		MDA-MB-231	Cell-growth-inhibitory activity, antioxidant activity	Decreases the growth of cancer cells at a concentration of 1600 μg/mL and pH 8.6. Alkaline pH increases the cytotoxic potential of neem	[133]
		MCF-7	Immunomodulatory effect	Induces Th1 immune response as evidence of the secretion of IFN-gamma and increases the production of IgG2a antibody in immunized mice	[109]
6	Neem leaf glycoprotein	cervical cancer stage IIIB cells	Relieves tumor immune suppression	Inhibits the induction of indoleamine 2,3 dioxygenase, limits the expression CTLA4 on Tregs, and induces normal maturation of dendritic cells	[88]
7	Epoxyazadiradione	TNBC MDA-MB-231 and ER+ MCF-7 breast cancer cells	Cell apoptosis, antimetastatic, and antiangiogenic	Inhibits the expression of proangiogenic and prometastatic genes Cox2, OPN, VEGF, and MMP-9. Attenuates PI3K/Akt-mediated AP-1 activation	[134]
8	Copper oxide nanoparticles of neem (CuONPs)	MCF-7 and HeLa	Cell apoptosis	Decreases proinflammatory cytokine level and proapoptotic protein expression, generates ROS inside the cancer cells, and induces DNA fragmentation	[135]

**Table 2:** Comparative anticarcinogenic properties of neem extracts in gynecological cancers based on in vivo studies.

Neem Extract	Animal Model	Administration Protocol	Mechanism of Action	Results	Reference
**Breast Cancer**					
Ethanolic leaf extract	BALB/c female mice with 4T1 induced breast cancer	Intratumoral injections of 500mg/kg of the neem extract every 48 h for 4 weeks after the tumor developed	Suppression of c-Myc oncogene expression		[105]
Ethanolic leaf extract	Sprague Dawley female rats with NMU-induced carcinogenesis	4 mg/kg (p.o) daily, 4 weeks	Increases caspase expression and p53, Bax, and Bad proteins and decreases MAPK1, Bcl-2, cyclin D1, and Cdk 2 activity	Suppressed tumor progression	[106]
Aqueous leaf extract	Swiss mice and Balb/c mice after BTAA-induced carcinogenesis	1 unit/week, 4 weeks	Decreases Th1 immunity cells and Il-10 and increases NK cells and IFN-gamma	Enhanced immune response to tumor vaccine	[109]
Methanolic leaf extract and ethyl acetate leaf fraction	Sprague Dawley female rats with DMBA-induced carcinogenesis	1–10 mg/kg (p.o), three times/week, for 12 weeks	Increases apoptosis through increased Bcl-2, NF-κB, and estradiol expression and decreases SOD, CAT, and caspase-3 activity	Suppressed tumor progression	[108]
Aqueous leaf extract	Swiss female mice with Ehrlich carcinoma	1 unit/week (p.o.), 4 weeks	Immunomodulation	Suppressed tumor growth	[136]
Ethanolic leaf extract	Balb/c female mice with 4T1 xenograft	250.5 mg/kg twice per day for 4 weeks	Suppression of c-Myc oncogene expression		[105]
Leaf glycoprotein	Swiss female mice with Ehrlich carcinoma	0.25 mg daily for 4 weeks		Reduced tumoral volume	[137]
Leaf glycoprotein	Swiss female mice with Ehrlich carcinoma	25 µg (s.c.) one time per week, 4 weeks	Suppression of VEGF and VEGFR2 expression	Normalized angiogenesis and suppressed tumor growth	[138]
**Cervical Cancer**					
Mixture of neem limonoids and other components	Patients with LSIL and HPV 16 infection	Intravaginal application of praneem tablet or placebo for 30 days, excluding menstrual period	Elimination of HPV DNA	Improved cytological abnormalities and clinical symptoms, eliminated HPV infection in 60% of cases	[91]

## Abbreviations

AKT	protein kinase B
AMPK	AMP-activated protein kinase
Apaf-1	apoptotic protease activating factor 1
ATM-kinase	protein kinase ataxia-telangiectasia mutated
BALB/c	albino, laboratory-bred strain of the house mouse
Bax	B cell lymphoma-2 protein associated X protein
Bcl-2	B cell lymphoma-2 protein
BTAA	breast tumor associated antigen
CAT	chloramphenicol acetyltransferase
Cdc37	Hsp90 co-chaperone encoded by the CDC37 gene
Cdk2	cyclin-dependent kinase 2
CEA	carcinoembryonic antigen
CKI p21	cyclin-dependent kinase inhibitor 1
CTLA4	cytotoxic T-lymphocyte antigen 4
CuONPs	copper oxide nanoparticles
Cyclin D1	cell cycle regulatory protein
CYP 1A1	gene that encodes a member of the cytochrome P450 superfamily
DNA	dezoxiribonuicleic acid
EAD	epoxyazadiradione
EAF	ethyl acetate fraction
EGFR	epidermal growth factor receptor
ERK	extracellular signal-regulated kinases
HDAC-2	histone deacetylase 2
HeLA	human cervical cancer cell line
HL60	human leukemia cell line
HPV	human papilloma virus
HSF1	heat shock factor 1
HSP90	heat shock protein 90
H3K27Ac	modification to DNA packaging protein histone H3
IFN-γ	interferon gamma
IGF	insulin-like growth factor
JNK	c-Jun N-terminal kinase
keap1	Kelch-like ECH-associated protein 1
LD_50_	median lethal dose
LSIL	low-grade squamous intraepithelial lesion
MAPK1	mitogen-activated protein kinase 1
MAPKs	mitogen-activated protein kinase
MCF-7	breast cancer cell line estrogen dependent
MCL-1	induced myeloid leukemia cell differentiation protein
MDA MB-231	breast cancer cell line estrogen independent
MEX	methanolic extract of neem oil
MF	methanolic fraction
NF-κB	nuclear factor κB
NO	nitric oxide
NOS	nitric oxide syntethase
Nrf2	nuclear factor (erythroid-derived 2)-like 2
NTERA-2	human embryonal carcinoma cells
OVCAR4	Cellosaurus cell line
p53	protein 53
PARP	pharmacological inhibitors of the enzyme poly ADP ribose polymerase
PI3K	phosphoinositide 3-kinase
PP2a	phosphatase 2a
PTEN	phosphatase and tensin homolog gene
SKOV3	ovarian cancer cell line
SMAD	family of structurally similar proteins that are the main signal transducers for receptors of the transforming growth factor beta superfamily
SOD	superoxide dismutase
TGF-β1	transforming growth factor
Th1	T helper 1
TNF α	tumor necrosis factor
TNBC	triple negative breast cancer cells
ER+	positive for estrogen receptors
VEGF	vascular endothelial growth factor
WHO	World Health Organization
